# Proposing a Model of Proactive Outreach to Advance Clinical Research and Care Delivery for Patients Who Use Tobacco

**DOI:** 10.1007/s11606-022-07553-x

**Published:** 2022-04-26

**Authors:** Jessica L. Burris, Tia N. Borger, Timothy B. Baker, Steven L. Bernstein, Jamie S. Ostroff, Nancy A. Rigotti, Anne M. Joseph

**Affiliations:** 1 Department of Psychology, University of Kentucky, Lexington, KY USA; 2 Department of Medicine, University of Wisconsin School of Medicine and Public Health, Madison, WI USA; 3 Department of Emergency Medicine, Geisel School of Medicine at Dartmouth, Hanover, NH USA; 4 Department of Psychiatry & Behavioral Sciences, Memorial Sloan Kettering Cancer Center, New York, NY USA; 5 Department of Medicine, Harvard Medical School, MB Boston, USA; 6 Department of Medicine and Masonic Cancer Center, University of Minnesota, Minneapolis, MN USA

## Abstract

There are evidence-based treatments for tobacco dependence, but inequities exist in the access to and reach of these treatments. Traditional models of tobacco treatment delivery are “reactive” and typically provide treatment only to patients who are highly motivated to quit and seek out tobacco treatment. Newer models involve “proactive” outreach, with benefits that include increasing access to tobacco treatment, prompting quit attempts among patients with low motivation, addressing health disparities, and improving population-level quit rates. However, the definition of “proactive” is not clear, and adoption has been slow. This commentary introduces a comprehensive yet flexible model of proactive outreach and describes how proactive outreach can optimize clinical research and care delivery in these domains: (1) identifying the population, (2) offering treatment, and (3) delivering treatment. Dimensions relevant to each domain are the intensity of proactive outreach (low to high) and the extent to which proactive outreach activities rely on human interaction or are facilitated by information technology (IT). Adoption of the proposed proactive outreach model could improve the precision and rigor with which tobacco cessation research and tobacco treatment programs report data, which could have a positive effect on care delivery and patient outcomes.

Tobacco use is a principal threat to global public health. In the USA, the prevalence rate for current tobacco use in adults is 14%,^1^ and among adults who use tobacco, only 55% make a serious quit attempt each year.^2^ Furthermore, only 31% of smokers who tried to quit in the past year used evidence-based treatment,^2^ with only 5% using the “gold standard” of cessation counseling and pharmacological therapy.^2^ Importantly, deep-rooted inequities in access to and reach of tobacco treatment contribute to some disadvantaged groups and historically marginalized populations bearing a disproportionate burden of tobacco use.^3,4^ Although tobacco use has declined significantly over the past half century, quit rates have plateaued, and both metrics fail to meet Healthy People benchmarks for tobacco prevention and control success.^4^ Thus, tobacco use remains the top cause of preventable and early mortality in the USA.^3^

There are effective tobacco treatments^5–7^ and there have been many efforts to enhance tobacco treatment delivery in healthcare settings. Traditional treatment models can be described as reactive, that is healthcare providers offer to provide or refer patients for treatment only after patients affirm active tobacco use and express readiness to quit. Often, therefore, the patient is at least partially responsible for initiating the process of treatment delivery. Unfortunately, population-based studies indicate that while 60–70% of smokers want to quit,^8,9^ at any time only 69% of these patients are actually “ready to quit,” defined by willingness to quit in the next year.^8^ Thus, one limitation of traditional approaches is only highly motivated patients receive attention and quit assistance.^10,11^ Another negative consequence of traditional models may be that patients perceive providers as passive in their approach to tobacco cessation and conclude that tobacco treatment is ineffective. Moreover, implicit or explicit biases with respect to race, ethnicity, mental health, substance use, or socioeconomic status can impact providers’ adoption of clinical practice guidelines,^12,13^ and contribute to a longstanding problem of inadequate, disparate reach of evidence-based tobacco treatment to certain populations of smokers.^12,14,15^ For example, smokers who were Asian, American Indian/Alaska Native, Hispanic, older, or uninsured had lower rates of receiving advice to quit by a health professional than those in other groups.^2^ Under the current model, every year approximately 70% of smokers who receive medical care do not receive evidence-based tobacco treatment.^16^

In contrast to reactive treatment models, proactive treatment models take a population-based approach with the objective of extending tobacco treatment to all patients who use tobacco.^10,17,18^ Evidence suggests many advantages of proactive treatment such as prompting quit attempts among patients who initially report very low or ambivalent motivation to quit,^19,20^ addressing health disparities via equitable opportunity to receive evidence-based treatment,^17,21^ and improving quit rates at the population level.^18,22,23^ Proactive treatment models are designed to and appear to increase the impact of evidence-based tobacco treatment.^24^

Given their potential advantages, attention to and investigation of proactive treatment models (hereafter referred to as “proactive outreach”) have increased. However, the empirical literature contains inconsistency in language and is imprecise regarding the processes through which proactive outreach is achieved. “Proactive” is sometimes considered synonymous with “opt-out,” “population-based,” “default,” and/or “motivation agnostic.” While these terms may accurately represent some cases of proactive outreach, they insufficiently describe the details of proactive outreach (i.e., the who, what, when, where, and how). Prior descriptions and demonstrations of proactive outreach to patients who use tobacco have included certain details about the intervention (often the novel aspects), but frequently fail to cover the components of proactive outreach comprehensively. To advance clinical science related to development, evaluation, and implementation of proactive outreach, we propose a taxonomy and strategic model for proactive outreach in the context of tobacco treatment delivery in clinical settings. The adoption of this more comprehensive yet flexible model could contribute to the field by improving consistency in reporting tobacco cessation outcomes, facilitating data reproducibility, and improving methodological rigor.

The goal of this commentary is to identify the key domains in which proactive outreach for tobacco treatment can optimize clinical research and care delivery pertinent. The key domains are as follows: (1) identifying the target population, (2) offering treatment to all current users and potentially even recent quitters, and (3) delivering treatment (see Fig. 1). In addition to outlining some clinical strategies for each domain, we suggest two over-arching dimensions are super-imposed on the three domains. One is the *intensity* of proactive outreach, which ranges from low to high; another is the range of proactive outreach activities that are fully automated and facilitated by *information technology* (*IT*), as opposed to reliant on human *in-person contact*. We suggest that proactive outreach descriptions and evaluations include detail about the three domains, as well as the intensity and degree of IT support of the intervention for the domain(s). We maintain there is no single definition of “proactive outreach,” but believe the proposed practice of reporting would facilitate replication studies and clinical implementation, which could clarify which intervention components are most critical to patient care.

**Figure 1.**
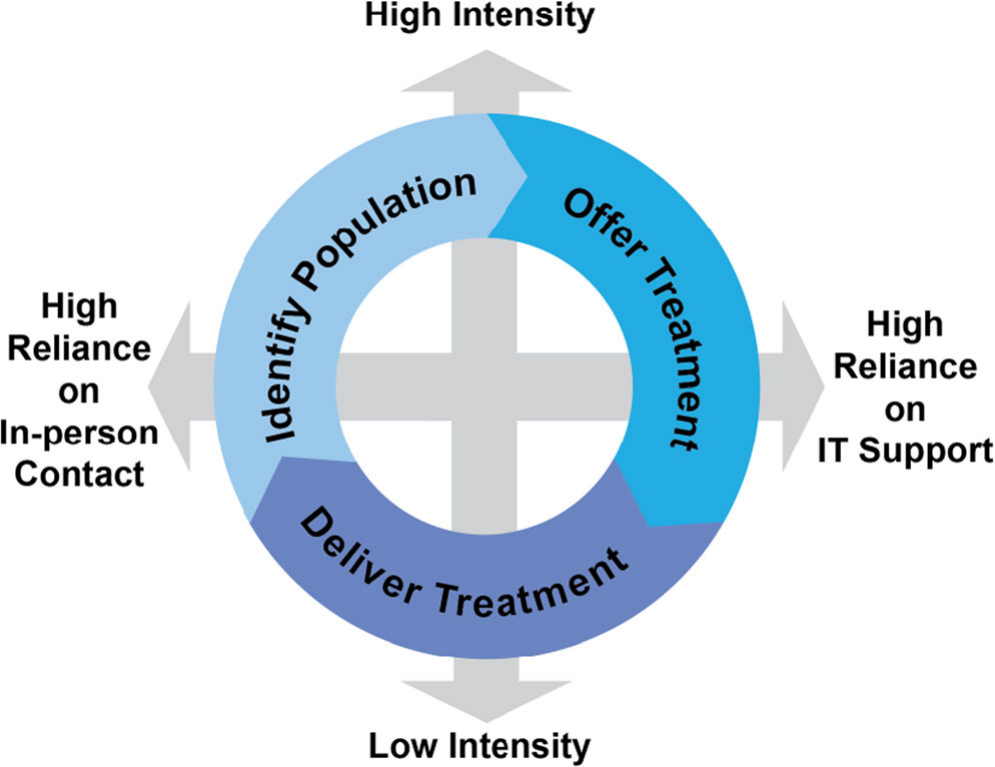
A model of proactive outreach to patients who use tobacco.

The proposed model acknowledges the reality that quit attempts often result only in short-term abstinence, or reductions in daily cigarette consumption, but not sustained quitting,^2^ and that patients often must cycle through treatment multiple times before quitting for good.^25,26^ Admittedly, many clinical trials and tobacco treatment programs currently include some (but not all) components of proactive outreach described below. Identifying the population in need and initiating treatment are essential to *reach* and treatment delivery is essential to *efficacy* and *effectiveness*; population health *impact* is a product of reach and efficacy in all three of these domains. To illustrate, a low-efficacy treatment that reaches a high proportion of tobacco users is likely to have a bigger population impact than a high-efficacy treatment that reaches only a small proportion of tobacco users.

## Identify Population

Definitions of the *population* that would benefit from proactive outreach vary considerably. Some programs target only patients scheduled for and attending a healthcare appointment, while others include anyone in a “tobacco user” registry created via the electronic health record (EHR). Other times the population is only those people who report tobacco use in electronic patient communications (e.g., an EHR-facilitated survey). The population may also include anyone with a certain diagnosis (e.g., cancer)^21^ or who is seen in a specific healthcare setting or point of service (e.g., hospital admission, lung cancer screening, outpatient surgery).^27^ Sometimes, patients must indicate readiness to quit in the next 1–6 months to be eligible for treatment, but sometimes the population is expanded to include people who are not ready to quit or who quit recently but remain at risk for relapse. “Tobacco user” can be defined as any form of tobacco, or specified as combusted cigarettes, electronic nicotine delivery systems, or smokeless tobacco, pipes, and/or cigars, with assessment and treatment tailored to the form of tobacco. Thus, the targeted population may be specific to an aspect of the patient’s motivation or behavior, diagnosis, clinic, health system, insurance type, or truly population based.

## Offer Treatment

There are many ways to proactively provide access to and engage patients in treatment. Some treatment referrals rely on human action, and some on IT. At one end of the spectrum, a staff person might distribute an educational booklet to everyone at check-in, or a provider may place a referral order during an exam. At the other end of the spectrum, an IT-supported system could automatically place an electronic referral to a quitline^28^ or activate a completely “no touch” interactive voice response (IVR) system.^29,30^ Tobacco treatment programs often rely on hybrid models for patient referral and engagement. For example, the EHR may place an automatic referral to a tobacco treatment specialist who telephones the patient to make recommendations for pharmacotherapy and provides motivational interviewing.^31^ In some cases, treatment offers are persistent; in others, patients have the option of declining early in the process. These decision points have large effects on reach of tobacco treatment.

## Deliver Treatment

The goal of proactive outreach could differ across patients, treatment programs, or clinical settings, and the goal has direct bearing on the treatment strategies chosen*.* For example, the clinical objectives might be to have patients make a first or repeat quit attempt, to try a pharmacotherapy or other evidence-based treatment to assist a quit attempt, to reduce the amount of tobacco use or even switch tobacco products in preparation for quitting, or to become interested in quitting.^25,32^ Some clinical strategies for these scenarios include brief advice, medication sampling, motivational interviewing, intensive individual or group counseling, quitline or other telephone-based counseling, texting programs, smartphone apps, Web/online programs, and IVR systems, all with varying treatment efficacy.^26,28–30^ Adherence to principles of shared decision making should result in greater reach and effectiveness.^18,22^

## Overarching Dimensions

**Figure 2.**
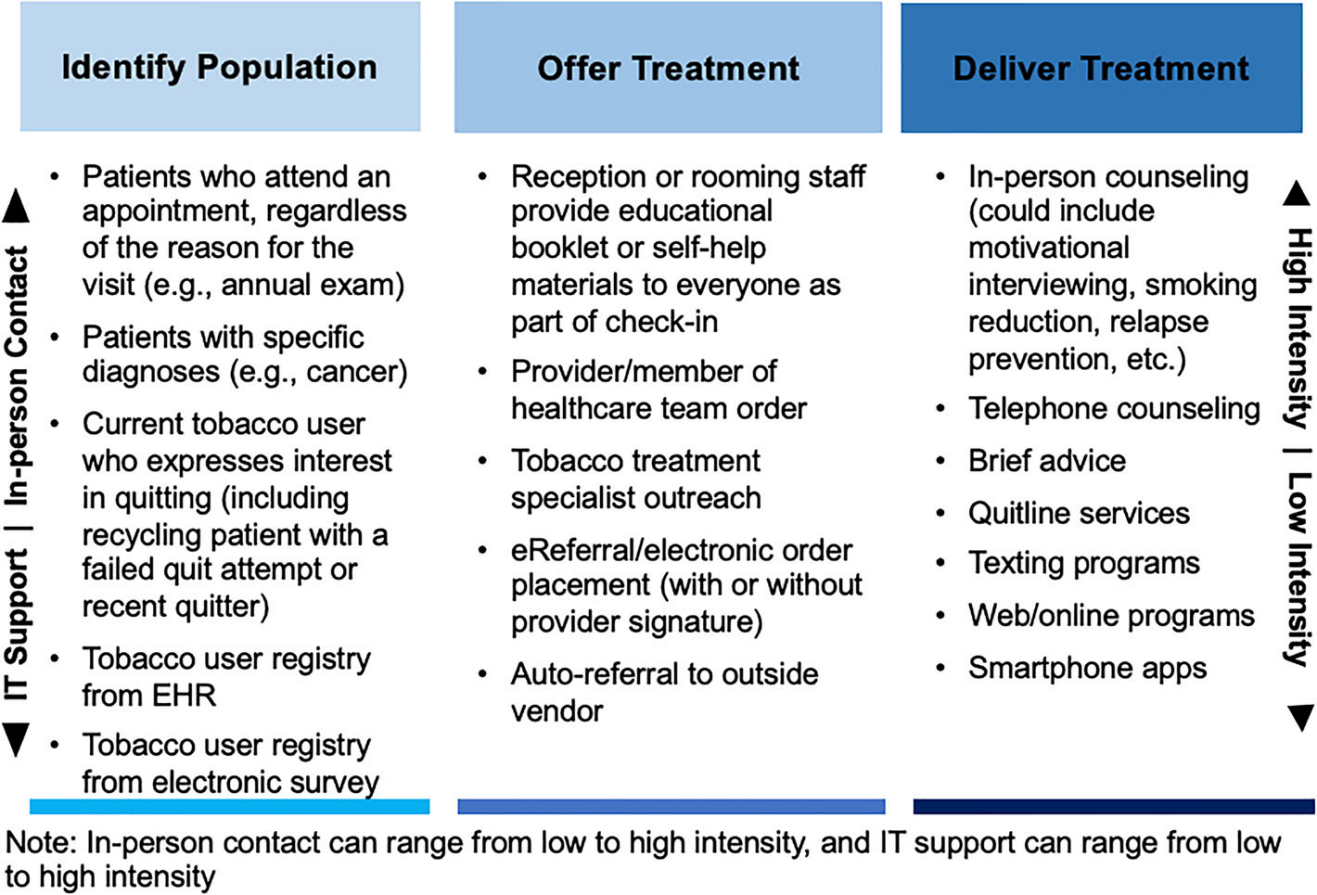
Scaled description of two dimensions that underlie the domains of proactive outreach.

For each domain of proactive outreach, Figure 2 provides a scaled description of the intensity continuum and human-to-IT continuum. This figure is not intended to be exhaustive, but rather to highlight the importance of describing these dimensions of a tobacco treatment program. The model points out that many aspects of proactive outreach require IT infrastructure to be effective, which requires healthcare system investment.

With respect to identification of populations, one strategy might be to assess tobacco use only at an annual “well visit” (low intensity), another might send a quarterly online survey (moderate intensity), and another might assess tobacco use at every visit, regardless of the reason for the visit (high intensity). A treatment offer might be defined by automatic placement of an electronic referral (low intensity), or an electronic referral followed by patient navigation via phone to ensure the patient schedules and attends an appointment (high intensity). Finally, treatment delivery might consist of IVR calls or text messages (low intensity) or multiple sessions of individual, in-person counseling with arrangement of pharmacotherapy support and IVR-based outcome assessment (high intensity). For each domain, there is a range of strategies that span those based on face-to-face interactions to those heavily supported by IT. There are inherent trade-offs along this spectrum, often between reach and efficacy. For example, reach may be lower for provider-initiated encounters and higher for technology-supported outreach activities.^33,34^ There are also resource trade-offs, for example high for human interactions and low for IVR; however, IVR may be less effective than counseling from a trained provider.^35–37^

## Recommendations

To advance tobacco treatment, we recommend the design and report of clinical trials and treatment programs that use proactive outreach models accurately detail how they (1) identify the population (e.g., reliance on a tobacco use “registry” from data captured via patient self-report, which could involve clinic personnel or IT support), (2) offer treatment (e.g., discipline and location of healthcare person responsible, whether the mechanism of referral requires a signature or approval or is completely generated by IT), and (3) deliver treatment (e.g., specific treatment components and modalities such as telephone coaching, texting, prescription, and delivery of pharmacological treatment). Furthermore, we suggest each domain be described with respect to level of intensity and reliance on human interactions and/or IT support. Guidelines and checklists for reporting clinical trials, such as TiDIER, might request information about each of these three domains for studies that test proactive outreach for tobacco treatment as well as other conditions.

This comprehensive model is novel because it situates the idea and practice of “proactive outreach” within a framework that allows specificity in measurement, description, and evaluation, and also acknowledges that varying levels of intensity and reliance on human/IT support could affect both treatment reach and effectiveness in complex ways. Future lines of research might focus on the cost effectiveness of various proactive outreach approaches, alone and in combination; strategies to optimize and sustain implementation; and patient, provider, and staff preferences for these approaches. Study designs like the Multiphase Optimization Strategy (MOST) that allow simultaneous testing of various treatment components might be particularly useful.^38^ Increasing patient engagement in tobacco treatment with greater adoption of proactive outreach is hypothesized as essential for achieving the full population benefit of evidence-based tobacco treatment and ultimately reducing the burden of tobacco-related disease.
